# Taxonomy and systematics of plant probiotic bacteria in the genomic era

**DOI:** 10.3934/microbiol.2017.3.383

**Published:** 2017-05-31

**Authors:** Lorena Carro, Imen Nouioui

**Affiliations:** School of Biology, Newcastle University, Newcastle upon Tyne, UK

**Keywords:** phylogeny, plant, probiotic, PGPR, IAA, ACC, genome, metagenomics

## Abstract

Recent decades have predicted significant changes within our concept of plant endophytes, from only a small number specific microorganisms being able to colonize plant tissues, to whole communities that live and interact with their hosts and each other. Many of these microorganisms are responsible for health status of the plant, and have become known in recent years as plant probiotics. Contrary to human probiotics, they belong to many different phyla and have usually had each genus analysed independently, which has resulted in lack of a complete taxonomic analysis as a group. This review scrutinizes the plant probiotic concept, and the taxonomic status of plant probiotic bacteria, based on both traditional and more recent approaches. Phylogenomic studies and genes with implications in plant-beneficial effects are discussed. This report covers some representative probiotic bacteria of the phylum *Proteobacteria*, *Actinobacteria*, *Firmicutes* and *Bacteroidetes*, but also includes minor representatives and less studied groups within these phyla which have been identified as plant probiotics.

## 1. What is a Plant Probiotic?

The concept of probiotic was first described by Elie Metchnikoff in the early 20^th^ century, in an attempt to identify some beneficial bacteria that could colonize the human gut. Today, probiotics are still associated with gut microbiota, although the FAO/WHO Expert Consultation Report defines them as “live microorganisms which when administered in adequate amounts confer a health benefit on the host” [1]. This definition is perfectly applicable to microorganisms responsible for improving plant development or protection against pathogens, but it has not been used in this sense until recently. The microorganisms able to live inside healthy plant tissues are called endophytes, they have a strong relationship with their host and, in most cases, this relationship is the response of millions of years of coevolution [2]. Indeed, plants are thought to rely on their microbiomes for faster adaptations to sudden environmental changes. While plants are quite limited in terms of adaptation (due to their inability to move and their slow mutation rate), microorganisms can compensate by evolving functionality more quickly with their short life cycles [2].

The plant probiotics concept includes all the microorganisms, specially fungi and bacteria—known as plant growth promoters (PGP)—due to their beneficial role in the general growth of plants and their faster adaptation to environmental changes, such as drought, heat or salinity. These microorganisms encompass the well-studied nitrogen suppliers (rhizobia strains or *Frankia*), other nutrient suppliers (*Pseudomonas*, which supply phosphorus), those that induce systemic resistance (*Trichoderma*) and those which directly protect the plants against pathogens (such as *Bacillus* spp. which produce fungicides). This review is focused on plant probiotic bacteria and their taxonomy.

Several bacterial genes are already known to be implicated in beneficial effects observed on plants, such as (i) genes involved in the fixation of atmospheric nitrogen (*nif* genes, which encode for the nitrogenase complex and other regulatory proteins); (ii) nodulation (*nod*); (iii) pathogen control (*chi* genes, which produce chitinases and *sfp* genes, which produce surfactins); (iv) phytohormone production (*acd*S, which encode the production of an ACC deaminase that improve tolerance to stress decreasing ethylene levels in the plant; and *ipd*C/*ppd*C implicated in indol acetic acid production); (v) vitamin production (*pqq*, which encode for pyrroloquinoline quinone) and (vi) nutrient mobilization (*bgl*/*ybg* genes, which are implicated in phosphate solubilization and *rhb* genes, which encode for siderophore production). Moreover, the implication of other genes from plant probiotics start to become known in the last years thanks to the new technologies available, as detailed below.

## 2. Classical Taxonomy and Systematics in Probiotics

### 2.1. Microbial classification

Taxonomy is a branch of biology that was established in the early 19^th^ century, but is a field on decline owing to the fact that the direct application of research is an imperative nowadays [3]. Many of the articles that can be found on the subject of taxonomy in probiotics are related to *Lactobacillus* strains and human probiotics [4],[5],[6], but there is limited information offered regarding the taxonomy of plant probiotics as a group. This is probably due to the high variability within this group of bacteria, which belong to several phyla, and because most of their phylogenetic analyses have been conducted within their specific genus, as can be seen in Supplementary Table 1.

**Table 1.** List of genera with confirmed capacity as plant probiotics.

Bacteria	Plant	References
***Phylum Actinobacteria***		
*Agromyces*	*Oryza sativa*	Bal et al., 2013 [146]
*Arthrobacter*	*Triticum aestivum*	Upadhyay et al., 2012 [147]
	*Brassica, Hordeum vulgare*, weed	Kim et al., 2011 [148]
*Curtobacterium*	Weed	Kim et al., 2011 [148]
	*Hordeum vulgare*	Cardinale et al., 2015 [149]
*Frankia*	*Atriplex cordobensis, Colletia hystrix, Trevoa trinervis, Talguenea quinquenervia, Retanilla ephedra*	Fabri et al., 1996 [150]
*Kocuria*	*Vitis vinifera*	Salomon et al., 2016 [151]
*Microbacterium*	*Hordeum vulgare*	Cardinale et al., 2015 [149]
	*Oryza sativa*	Bal et al., 2013 [146]; Banik et al., 2016 [152]
	*Arabidopsis thaliana*	Schwachtje et al., 2012 [153]
	*Vitis vinifera*	Salomon et al., 2016 [151]
	*Brassica*, weed	Kim et al., 2011 [148]
*Micromonospora*	*Lupinus angustifolia*	Trujillo et al., 2010 [13]; Trujillo et al., 2015 [154]
	*Discaria trinervis*	Solans, 2007 [155]
*Streptomyces*	*Aristida pungens, Cleome arabica, Solanum nigrum, Panicum turgidum, Astragallus armatus, Peganum harmala, Hammada scoparia, Euphorbia helioscopia*	Goudjal et al., 2014 [156]
	*Triticum aestivum, Solanum lycopersicum*	Anwar et al., 2016 [157]
	*Discaria trinervis*	Solans, 2007 [155]
*Rhodococcus*	*Oryza sativa*	Bertani et al., 2016 [41]
	*Hordeum vulgare*, weed	Kim et al., 2011 [148]

***Phylum Bacterioidetes***		
*Flavobacterium*	*Triticum aestivum*	Gontia-Mishra et al., 2016 [158]
	*Capsicum annuum*	Kolton et al., 2014 [159]
	*Pholidota articulata*	Tsavkelova et al., 2007 [160]
	*Solanum lycopersicum*	Subramanian et al., 2016 [161]
*Chryseobacterium*	*Glycine max*	Simonetti et al., 2015 [162]
	*Pholidota articulata*	Tsavkelova et al., 2007 [160]
	*Vigna unguiculata*	Leite et al., 2017 [49]
*Pedobacter*	*Solanum lycopersicum*	Subramanian et al., 2016 [161]
*Sphingobacterium*	*Vigna unguiculata*	Leite et al., 2017 [49]

***Phylum Firmicutes***		
*Bacillus*	*Triticum aestivum*	Upadhyay et al., 2012 [147]
	*Allium cepa, Allium fistulosum, Allium sativum, Brassica, Hordeum vulgare*, weed	Kim et al., 2011 [148]
	*Oryza sativa*	Bal et al., 2013 [146]; Banik et al., 2016 [41]
	*Glycine max*	Simonetti et al., 2015 [162]
*Brevibacillus*	Weed	Kim et al., 2011 [148]
	*Oryza sativa*	Bertani et al., 2016 [41]
*Lysinibacillus*	Weed	Kim et al., 2011 [148]
*Paenibacillus*	*Allium fistulosum, Brassica napa, Hordeum vulgare*, weed	Kim et al., 2011 [148]
	*Oryza sativa*	Bal et al., 2013 [146]; Banik et al., 2016 [152]
*Sporosarcina*	Weed	Kim et al., 2011 [148]
*Terribacillus*	*Vitis vinifera*	Salomon et al., 2016 [151]
*Viridibacillus*	Weed	Kim et al., 2011 [148]

***Phylum Proteobacteria***		
*Acetobacter*	Weed	Kim et al., 2011 [148]
*Achromobacter*	*Capsicum annuum*	Kong et al., 2016 [163]
*Acinetobacter*	*Allium cepa*	Kim et al., 2011 [148]
	*Oryza sativa*	Banik et al., 2016 [152]
*Aeromonas*	*Oryza sativa*	Banik et al., 2016 [152]
*Agrobacterium*	*Pholidota articulata*	Tsavkelova et al., 2007 [160]
*Aminobacter*	*Anthyllis vulneraria*	Maynaud et al., 2012 [164]
*Ancylobacter*	*Oryza sativa*	Banik et al., 2016 [152]
*Azospirillum*	*Lactuca sativa*	Fasciglione et al., 2011 [165]
	*Zea mays*	Couillerot et al., 2013 [166]
	*Oryza sativa*	Chaman et al., 2013 [167]; Banik et al., 2016 [152]
*Azorhizobium*	*Oryza sativa*	Banik et al., 2016 [152]
*Azotobacter*	*Oryza sativa*	Banik et al., 2016 [152]
*Bradyrhizobium*	*Lupinus albus*	Quiñones et al., 2013 [168]
	*Oryza sativa*	Banik et al., 2016 [152]
*Burkholderia*	*Paphiopedilum appletonianum*	Tsavkelova et al., 2007 [160]
	Weed	Kim et al., 2011 [148]
	*Oryza sativa*	Bertani et al., 2016 [41]; Banik et al., 2016 [152]
*Devosia*	*Neptunia natans*	Rivas et al., 2002 [169]
*Ensifer*	Weed	Kim et al., 2011 [148]
	*Oryza sativa*	Banik et al., 2016 [152]
	*Psoralea corylifolia*	Prabha et al., 2013 [170]
*Enterobacter*	*Triticum aestivum*	Gontia-Mishra et al., 2016 [158]
	*Oryza sativa*	Banik et al., 2016 [152]
*Erwinia*	*Paphiopedilum appletonianum*	Tsavkelova et al., 2007 [160]
*Herbaspirillum*	*Oryza sativa*	Bertani et al., 2016 [41]
*Janthinobacterium*	*Capsicum annuum*	Kong et al., 2016 [163]
*Klebsiella*	*Triticum aestivum*	Gontia-Mishra et al., 2016 [158]
	*Oryza sativa*	Banik et al., 2016 [152]
*Kosakonia*	*Oryza sativa*	Bertani et al., 2016 [41]
*Marinobacterium*	*Psoralea corylifolia*	Sorty et al., 2016 [171]
*Mesorhizobium*	*Cicer arietinum*	Brígido et al., 2016 [172]
	*Leucaena leucocephala*	Rangel et al., 2017 [173]
*Methylophaga*	*Oryza sativa*	Bal et al., 2013 [146]
*Methylotropic*	Weed	Kim et al., 2011 [148]
*Methylobacterium*	*Oryza sativa*	Bertani et al., 2016 [41]
*Novosphingobium*	*Oryza sativa*	Banik et al., 2016 [152]
*Pantoea*	*Oryza sativa*	Bertani et al., 2016 [41]; Banik et al., 2016 [152]
	*Allium fistulosum*	Kim et al., 2011 [148]
*Phyllobacterium*	*Arabidopsis thaliana*	Kechid et al., 2013 [174]
*Pseudomonas*	*Allium cepa, Allium fistulosum, Brassica napa, Hordeum vulgare, weed*	Kim et al., 2011 [148]
	*Arabidopsis thaliana*	Schwachtje et al., 2012 [153]
	*Hordeum vulgare*	Cardinale et al., 2015 [149]
	*Glycine max*	Simonetti et al., 2015 [162]
	*Oryza sativa*	Bertani et al., 2016 [41]; Banik et al., 2016 [152]
	*Solanum lycopersicum*	Subramanian et al., 2016 [161]
	*Mentha piperita*	Santoro et al., 2016 [175]
*Ochrobactrum*	*Oryza sativa*	Pandey et al., 2013 [176]
	*Arachis hypogaea*	Paulucci et al., 2015 [177]
*Ralstonia*	*Oryza sativa*	Bertani et al., 2016 [41]
*Ranhella*	Weed	Kim et al., 2011 [148]
*Rhizobium*	*Capsicum annuum, Lycopersicon esculentum*	Garcia-Fraile et al., 2012 [178]
	*Oryza sativa*	Banik et al., 2016 [152]
	*Psoralea corylifolia*	Prabha et al., 2013 [170]
*Serratia*	*Allium fistulosum*	Kim et al., 2011 [148]
*Stenotrophomonas*	*Hordeum vulgare*	Kim et al., 2011 [148]
	*Capsicum annuum*	Kong et al., 2016 [163]
	*Oryza sativa*	Banik et al., 2016 [152]
	*Pholidota articulata*	Tsavkelova et al., 2007 [160]
*Staphylococcus*	*Solanum melongena, Capsicum annuum*	Amaresan et al., 2014 [179]
*Variovorax*	Weed	Kim et al., 2011 [148]

First microbial classifications rely on phenotypic characterization of isolated strains, from morphological aspects to biochemical identification, trying to identify the functional capabilities through culture-based methods. However, these tests alone induced many taxa misclassifications that could not be solved until the introduction of genetic features and polyphasic approaches [7].

### 2.2. Genotyping approaches

Following the isolation of potential plant probiotics, analyses used to include genotyping characterization of strains, including methods such as RAPD (Random Amplification of Polymorphic DNA) [8], ERIC-PCR (Enterobacterial Repetitive Intergenic Consensus) [9], BOX-PCR (Repetitive extragenic palindromic sequences) [10], RFLPs (Restriction Fragment Length Polymorphism) or ARDRA (Amplified rDNA Restriction Analysis) [11] to classify the microorganisms obtained [12],[13],[14]. These kinds of analyses are useful for grouping the bacteria into phylogenetic clusters. The first three methods determine clones within the isolated strains whilst the last two give information at species level. However, it has also been shown that the presence of genes related with promotion characteristics are strain-dependent, rather than the species or higher-level taxonomic group to which they belong [14].

With the appearance of the gold-standard marker in microbiology, the 16S rRNA gene, the identification of the strains has become feasible at genus and species level. This gene was selected owing to specific characteristics, such as size, ubiquity in bacteria, and slow rates of evolution. An in-depth analysis of bacteria phylogeny has incorporated “housekeeping genes”, using the multilocus sequence analysis (MLSA) approach. The selected genes depend on the group of bacteria, since several studies have shown that certain genes are more efficient in some genera than in others. For example, the gene which codes for recombinase (*rec*A), has been shown to be able to properly differentiate strains of the genus *Rhizobium* [15], while its use is relatively poor in the *Micromonospora* genus [12]. Typical “housekeeping genes” used in phylogeny include: *atp*D (ATP synthase subunit beta), *dna*J (chaperone dnaJ or heat shock protein 40 kD), *dna*K (chaperone dnaK or heat shock protein), *gap* (glyceraldehyde-3-phosphate dehydrogenase), *gln*A (glutamine synthetase), *glt*A (citrate synthase), *gyr*A (DNA gyrase subunit A), *gyr*B (DNA gyrase subunit B), *hsp*60 (heat shock protein 60 kD), *hsp*65 (heat shock protein 60 kD), *inf*B (translational initiation factor), *phe*S (phenylalanyl-tRNA synthase alpha subunit), *pnp* (polynucleotide phosphorylase), *rpo*A (RNA polymerase alpha subunit), *rpo*B (RNA polymerase beta subunit), *rec*A (recombinase), *tru*A (tRNA pseudouridine synthase A) or *thr*C (threonine biosynthesis gene C) [16]–[24].

The taxonomic status of a strain is not restricted to molecular methods, and both phenotypic and genetic characterization also needs to be performed according to the polyphasic taxonomy [7]. This characterization calls for combining chemotaxonomic features with phenotypic ones, such as enzyme production, tolerance tests (Temperature, NaCl concentration or pH), antibiotic resistance, ability to metabolize carbon and nitrogen sources, in addition to other genetic traits of the taxon such as GC content and DNA-DNA hybridization (DDH) with the closest type strains. In fact, prokaryotic species delineation was defined by a group of strains sharing more than 97% of 16S rRNA similarity [25], 70% of DDH [26], and/or 95–96% of average nucleotide identity (ANI) [27],[28]. For plant probiotic bacteria, the complete polyphasic approach is determined for most of the studies only when the probiotic strains seem to represent a new species of their genus, while the majority of strains isolated lack of these analyses.

Recently, next generation sequencing (NGS) technologies have become everyday tools for laboratories, with hundreds of genomes and metagenomes generated each day [29]. The application of this information to different fields, such as plant probiotic analysis or taxonomic determination, is still in its first stages. We will analyze here how these technologies are applied to the study of some taxa described as plant probiotics.

## 3. Next Generation Sequencing

The evolution of next generation sequencing (NGS) has vastly increased our ability to obtain full genome sequences of prokaryotes in a scale of cost and time never seen before, making great strides both economically and technically. The use of NGS techniques permits the analysis in parallel of different molecules, genes using genomics, transcripts using transcriptomics, proteins using proteomics and metabolites using metabolomics [30]. This genomic information will change the approach of many biological disciplines owing to new information being obtained in a more easy and reliable manner, as has happening with the understanding of human microbiome effects [31] or unravel the brain complexity [32]. The study of gene composition in a bacterial genome or in the comparative analysis between bacteria with specific functions or ecological roles looking for differences allow a better comprehension of host-microbe interactions [33]. Within these interactions, one of the first questions tried to be answered using NGS technologies was “Who is there?” using the metagenomic analysis of plant samples [30]. The technologies applied in understanding plant microbial communities and their interactions has been studied in detail in previous reviews, from library preparation [34] to metabolic engineering by gene edition [35].

## 4. Metagenomic Analysis and Diversity

Probiotic bacteria have attracted the attention of the scientific community due to their beneficial effects on human, plant and animal health [36],[37]. One of the reasons for the increasing use of probiotic concepts in plant-microorganism relationships has been the development of these new sequencing technologies—mainly metagenomics analysis—which has recently highlighted the complex composition of symbioses, showing a rich microbial community living together within healthy plants [38],[39],[40]. Most of these studies describe the presence of *Proteobacteria, Firmicutes, Bacteroidetes*, and *Actinobacteria* as the most abundant phyla detected [39],[41]. All of these groups contain strains that have been described as plant growth promoter bacteria (PGPB), e.g. *Azospirillum* or *Rhizobium* in *Proteobacteria*, *Bacillus* or *Paenibacillus* in *Firmicutes*, *Flavobacterium* or *Pedobacter* in *Bacteroidetes*, and *Streptomyces* or *Micromonospora* in *Actinobacteria* (Table 1). The abundance of microorganisms belonging to the phyla *Proteobacteria* has been highlighted in most of the studies determining plant microorganism´s diversity, with a range from 40 to 90% for either isolation or microbiomes analyses [41],[42],[43]. However, these percentages are different depending on the part of the plant analyzed, with high variations between roots, nodules, stems, leaves or flowers [41],[43],[44]. *Proteobacteria* is the largest prokaryotic phylum [45] and is commonly found in soil and water habitats; therefore, it is expected that its presence in plants will be high but in many occasions only represent an incidental event rather than a symbiotic relationship. However, there are also many genera that have never been previously studied in their relationship with plants (e.g. *Terracocus* genus [46]), as well as many that were unknown before these metagenomic analyses allowed their determination without previous isolation [40].

To understand how a plant's probiotic bacterium interacts with its host, it is also necessary to understand how it interacts with its plant microbiome, a system in which complex interactions can be analyzed [47]. The compounds produced through plant-bacteria interactions are also implicated, as has been shown for the jasmonic acid (JA), whose induction is related to plant defenses and is a selective characteristic for PGPR bacteria. Liu et al. [48] have analyzed how JA expression could have an influence on the microbiome of wheat plants. The results showed that the presence of JA changes the root endophytic communities, reducing their diversity, but not changes were observed on shoot or rhizospheric communities. The analysis of the diversity of the microorganisms, and the taxonomic groups to which they belong, was made using Operational Taxonomic Units (OTUs), which are artificial groupings of taxa based on sequence similarity. These OTUs are used to determine the genus of the microorganisms analyzed, but can be used no further in the taxonomy due to the absence of a more complete genomic database and the inability to properly differentiate the data obtained into separated taxa.

The OTUs present in plant tissues where symbiosis occurs have been also analyzed in several legumes to determine their relationship with the symbiotic bacteria, the soil, and the plants [49],[50],[51]. However, each of these studies has highlighted that the main influence is carried out by a different agent. While analysis by Xiao et al. [51] found that plants determine the microorganisms detected as endophytes, Leite et al. [49] show that soils have an important influence on plant bacterial communities, higher than the plants themselves. On the other hand, the analysis presented by Zgadza et al. [50] remarks on the important influence of nodule symbiotic bacteria and their ability to fix nitrogen as key in the other endophytes selection. Changes in the plant microbiome generate perturbations in the balance of associated ecosystems. The understanding of these processes could be used in agriculture to induce specific changes or responses for plant health improvement [52].

General analysis of probiotic bacteria on plants are focused on the determination of their PGP characteristics; however, new analysis has shown that other parameters should also be of high importance when attempting to determine the necessary bacteria for the wellbeing of plants. Shade et al. [44] found that a phyla that is not used to take into account in these analyses, *Deionococcus-Thermus*, is highly abundant in plant flowers, and probably influence their development. Furthermore, the work of Ushio et al. [53] has shown how the flower microbial communities are signatures for pollinators, and thus, have a direct effect on plant reproduction. The study of plants and their microbiomes as a whole entity is key to understanding their ecology and understanding how small changes can affect the whole system balance.

## 5. Genome Analysis Looking for Promotion Genes of Plant Probiotic Bacteria

### 5.1. Proteobacteria

Analysis of individual genomes from plant probiotics in order to determine potential genes alongside their plant relationships is a major objective at this moment in laboratories worldwide [54]. Several recent studies are focused on the “rhizobia” group of bacteria, the nitrogen-fixing bacteria that lives in symbiosis with legume plants [55],[56]. In a study by Seshadri et al., [56], 163 of these genomes (44 of *Rhizobium*, 41 of *Ensifer*, 36 of *Bradyrhizobium*, 17 of *Mesorhisobium*, 13 of *Burkholderia*, 5 of *Cupriavidus*, 3 of *Methylobacterium*, 2 of *Azorhizobium* and 2 of *Microvirga*) have been analyzed and compared to non-nodule bacteria in order to identify specific genes of host-rhizobia interactions. Researchers have found that, beyond their ability for nitrogen fixation, most of these bacteria possess other capacities for plant growth promotion, such as 1-aminocyclopropane-1-carboxylate (ACC) deaminase (*acd*s), biocontrol or stress tolerance genes [56]. Most of the genome sequences of non-symbiotic plant probiotics available today belong to the *Pseudomonas* genera. The study of the *Pseudomonas fluorescens* F113 genome compared to 50 genomes of other *Pseudomonas* strains has revealed several specific characteristics specific to its plant interactions [57]. Several genes have been identified as potential probiotic genes, being implicated in motility, chemotaxis and antimicrobial compounds production. Recently, the inclusion of more PGP *Pseudomonas* strains has allowed the identification of more traits related with plant interaction [52]. Garrido-Sanz et al. [58] have determined that the presence of the genes for 2,4-diacetylphloroglucinol (DAPG); for pyoluteorin, for phenazine-1-carboxylic acid (PCA); and for phenazines or for pyrrolnitrin—all of them related with antifungal activity—are specifically related to precise clusters within the *Pseudomonas* strains. They have also detected genes related to the production of siderophores (to sequester iron from the environment), the production of indol-3-acetic acid (IAA) (a plant hormone related with growth and development), the degradation of phenylacetic acid (PAA) (a molecule that has been related with root colonization [59]), the synthesis of polyamine spermidine (related to a resistance to salinity, drought and cold temperatures in plants), and the denitrification process.

Within the same phyla, the genome analysis of 304 *Proteobacteria* to determine the presence of genes related with plant probiotic characteristics was carried out [33]. The presence of 23 genes was analysed over known PGPR, endophytic, saprophytic, and phytopathogenic bacteria, and Bruto et al. [33] proposed that the distribution of some of these bacteria could be related to certain taxonomic properties, as some distributions were according to their ecological type. Between the genes analyzed were those related to phosphate solubilization; pyrroloquinoline quinone (*pqq*BCDEFG); 2,4 diacetylphloroglucinal synthesis (*phl*ACB); indole-3-pyruvate decarboxylase/phenylpyruvate decarboxylase synthesis (*ipd*C/*ppd*C); hydrogen cyanide synthesis, acetoine/2,3-butanediol synthesis; nitric oxide synthesis (*nir*K); acetoine/2,3-butanediol synthesis (*bud*ABC); auxin synthesis (indole-3-pyruvate decarboxylase/phenylpyruvate decarboxylase gene (*ipd*C/*ppd*C); ACC deamination (*acd*s); and nitrogen fixation (*nif*HDK) [33]. In other proteobacteria studies, the presence of genes related to the production of siderophores, acetoin, butanediol, hydrogen sulfide (H_2_S), heat and cold shock tolerance, glycine-betaine production, or genes involved in oxidative stresses (catatases, peroxidases, and superoxide dismutases) has been demonstrated [60]. The genome analysis of an *Azospirillum* strain has also provided insights regarding plant hormone synthesis of this bacterium, and has shown high probability for another pathway for production of auxin [61]. In addition, thanks to horizontal gene transfer events, an adaptation of *Azospirillum* *amazonense* to the environment and its host plant has been proposed.

### 5.2. Actinobacteria, Bacteroidetes and Firmicutes

Much fewer studies related to PGP genes have been carried out on *Actinobacteria*, *Bacteroidetes* and *Firmicutes* phyla detected as plant endophytes. However, as many of their genomes start to become available [62]–[65], new studies to analyze these features are expected to be produced in the following years. Within the *Actinobacteria*, the complete genome of *Micromonospora* *lupini* Lupac 08, isolated from nitrogen-fixing root nodules of a legume plant, has been shown to possess characteristics of a PGP [66]. These characteristics were detected through genome mining and wet-lab techniques, indicating the production of several phytohormones, siderophores and defensin compounds. The presence of genes related to plant-polymer degrading enzymes was also detected, and a role in internal colonization was proposed for them [66]. Within the *Bacteroidetes*, 25 genomes of root-associated *Flavobacterium* were analyzed to identify markers for niche adaptation, and found that plant-related *Flavobacterium* could be determined by the presence of genes involved in the metabolism of glucans containing arabinose and rhamnogalacturonan [42]. Within the *Firmicutes*, the most abundant analyses are found for *Bacillus* genus. A comparative study using 31 genomes shows that plant-related *Bacillus* strains contain more genes related to intermediary metabolism and secondary metabolites production than those which are unrelated [67]. Most of the genome mining studies on *Bacillus* which have been presented so far are focused on secondary metabolites and antibiotic/antifungal compounds, due to their use as biocontrol agents [68],[69]. On the other hand, *Paenibacillus* genomic analysis has focused on other plant-growth promoting traits, such as nitrogen fixation, IAA production, or genes related to phosphate solubilization and assimilation [3].

The comparison between probiotic bacteria and their closest phylogenetic neighbors which are not able to colonize plant tissues, reveals necessary features for establishing and maintaining bacteria-plant interactions [47]. The information about plant-related bacteria genomes will be highly increased in the near future, thanks to the massive sequencing projects that are being carried out, and include the sequencing of genomes from soil and plant-associated bacteria [70].

## 6. Phylogenomic and Genomic Analysis Highlighting the PGPR Traits

### 6.1. Taxonomy in the genomic era

Taxonomy has a crucial role, not only in the classification and identification of, and differentiation between, the probiotic species or strains, but also in understanding the relationship between them and their habitats. Descriptions of a particular physiological or functional characteristic of species linked to plant-beneficial effects calls for an application of these bacteria in probiotic products. In addition, giving an appropriate name to each species or genus avoids confusion and allows for the taxon to be recognizable around the word.

In this scenario, traditional taxonomy based on the morphological and biochemical traits does not accurately distinguish between phenotypically-similar species and/or determine the phylogenetic relationship of some bacteria [71]. Modern taxonomy based on the single gene 16S rRNA gene, the MLSA, and the DDH, in addition to the emersion of several molecular typing methods, dramatically increases the number of the species or genus of bacteria with potential probiotic effect. However, all methods cited above have their own limitations which can be explained by (i) the low variability in the 16S rRNA gene sequence for some taxa, which handicap its efficiency in the differentiation of microorganism at species level; (ii) the dependence of the MLSA technique on the choice of the housekeeping genes, which differ from one taxa to another [72], in addition to their influence by horizontal gene transfer [73],[74]; and (iii) the time consuming technique of wet lab DDH, which is difficult to reproduce.

NGS have allowed overcoming some of these limitations with the availability of thousands of prokaryotic genome sequences and the accessibility to tools for phylogenomic analysis in public databases, with an important impact on the taxonomic community. Several methods have been developed to clarify the taxonomic status of some taxa which calls for a reexamination, since the phylogenetic analysis that the methods were based on were not sufficient [75]. These new methods based on genome information are helping to clarify the phylogeny of some microorganisms and provide a better understanding of their inter- and intra-relationships, and their evolutionary path. It has been shown that the phylogenomic approach has had a reliable impact on the phylogeny and the taxonomy of prokaryotes [76],[77]. Methods based on the complete genome sequence—as nucleotide composition relying on the tetranucleotide frequencies [78],[79], protein-encoding gene families [80],[81], and the gene order [82],[83]—have been used to highlight the relationship between microorganisms. Moreover, the gene-content approach reflecting the “pan genome” of species, the “core genome” (for all the strains), the “dispensable genome” (only for some strains), and the “unique genes” (for specific strains) are powerful methods in taxonomic classification [83].

### 6.2. Analyses in Proteobacteria

Comparative genomic approaches have led to the detection of significant markers for taxonomic classification, and the revelation of several genes with plant-growth-promoting functions in bacteria, which were not previously recognized as PGPR [38]. Between these genes appear *phl*ABCD, *pqq*FG, *bud*ABC, *ipd*C, *ppd*C and *hcn*ABC, which have been shown to have a strong relationship with proteobacterial phylogeny [33], while other PGPR genes appear to have weaker phylogenetic signals. To take advantage of PGP microorganisms with high confidence in their effectiveness in enhancing plant growth, their taxonomic status and their phylogenetic relationship need to be studied based on a state-of-art of genomic analysis. Genome and phylogenetic relationships analyses of plant probiotic bacteria have also been shown to be important in the determination of their evolution and the timing for colonization of terrestrial and plant habitats [84]. Moreover, it has been shown that phylogenomics is a powerful approach for an ideal taxonomic affiliation of taxa with a very low risk of mislabeling.

Whole genome sequences were used for the classification and analysis the PGPR effect of three rhizobacteria isolated from a commercial plantation [60]. The phylogeny of the isolates and their relatedness to the other group of PGPR were determined using pairwise genome comparison, showing the membership of 2 isolates to *Enterobacter cloacae* [85],[86] and one to *Pseudomonas putida* [87]. Screening the genome of these three rhizobacteria for the PGP genes led to the detection of common genes such as catalases, superoxide dismutase, peroxidases and glutathione transferases, all involved in the protection of the plant against oxidative stress, and hydrogen sulfide (H_2_S), known recently by its increasing effect on seed germination. Moreover, different genes with the same function as *rimM*, *dcy*D (*Enterobacter* group) and *acd*S (*Pseudomonas* groups), all of which encode for ACC deaminase, have been detected. However, other genes, as *fpv* and *mbt*, encoding for the same function, pyverdine production, seem to be restricted to the taxonomic genus rank, because they are only present in the genomes of isolates belonging to the genus *Enterobacter*. Conversely, other molecular markers were found to be present to the species or strain level as *bud*AC [88] and *als* [89] at the origin of 2,3-butanediol and acetoin production, respectively. These were later detected in only one of the isolates belonging to *Enterobacter* group [60]. The comparison of PGP rhizobia with the closest neighbor non-PGPR taxon provides important genetic information regarding the PGP properties of some strains or species. In this regard, Gupta et al. [60] has shown that *P. putida*, which is well known by its PGP traits, lacks some PGP genes encoding for enterobactin, siderophore, pyrroloquinoline quinone and phenazine biosynthesis. The comparison of 50 genomes of *Pseudomonas fluorescens* strains analyzed in the previous section shows a classification of five main subgroups that possess a highly conserved core genome, but probably should be divided into five separate species [57]. Indeed, an update on the evaluation of this *P. fluorescens* complex has been presented recently, including the DDH *in silico* with several methods, and shows that hundreds of species compose this complex [58]. More recent analysis of this genus (but in this case using plant pathogenic *Pseudomonas*) has shown how genome similarity can be used in taxonomy to analyze strains that have been considered the same species until now, establishing differences within the pathovars analyzed and using a proposal that can be extended to other bacteria [90].

Among the taxa of the phylum *Proteobacteria*, the genus *Azospirillum* was recognised as PGPB due to their beneficial effects on plants. The genus encompasses 19 species with validly published names, according to LPSN classification [91]. The particular taxonomic status of the *Azospirillium amazonense* species, which showed a closer phylogenetic relationship to *Rhodospirillum centenum* and *Azospirillum irakense* than to *Azospirillum brasilense*, and distinctive phenotypic and physiological features which increase its beneficial role in PGP compared to the other member of the genus *Azospirillum*, called for comparative genomic of *A. amazonense* with its closely phylogenetic neighbor. Genomic information has highlighted a number of specific genes for *A. amazonense* which are not common with any other *Azospirillium* species but are more related to *Rhizobiales* [61].

The genomic screening of 23 genes recognized as PGP, based on 304 genome sequences of proteobacteria and non-PGPR proteobacteria, showed a taxonomic specific link between the bacteria and their ecological type: saprophyte, symbiotic, endophyte, plant and animal pathogens. In fact, the following PGP gene *phl*ACB was restricted to only 3 proteobacteria genomes, while *ppd*C, was detected in some *Azospirillium* and *Bradyrhizobium*, which belong to endophyte/symbiont category. Genes *nif*HDK were retrieved in different symbiont endophytes of proteobacterial taxa and bacteria classified as PGPR, while genes *hcn*ABC (hydrogen cyanide synthesis) were detected in all non-phytopathogenic *Pseudomonas* strains [92]. However, several genes were found to be distributed independently of the taxonomic rank, which is the case for *nir*K and *pqq* genes propagated in different *Proteobacteria. Alphaproteobacteria*, *Gammaproteobacteria* and all members of *Burkholderiaceae* contained *acd*S, while *ipd*C and *bud*AB genes were mainly present in *Enterobacteteriaceae* [92]. Within this study is apparent that analyzing PGP gene occurrence helps to determine the lifestyle boundaries at species and strain level [92]. In this context, the genus *Burkholderia* encompasses 103 species from different ecological niches, with pathogens to humans, animals or plants in additional to the environmental species. Among the last ones, some members were identified as plant growth promoting bacteria, such as *Burkholderia phytofirmans* [93]. This latter category of *Burkholderia* was classified into a new genus—*Paraburkholderia*—after the taxonomic revision of this genus based on the phylogenomic and comparative genomic analyses [94]. Using a concatenated tree based on 21 conserved proteins for 45 species covering the genetic diversity of the genus, and the 16S rRNA gene, the genus was structured in two superclades in which the clinical species, *Burkholderia cepacia* complex (BCC), and *Burkholderia pseudomallei*, were grouped together in distinct clades from the environmental species. These findings were in line with the comparative genomic analysis which led to the detection of six specific conserved protein sequences for pathogenic *Burkholderia* and 2 for the environmental species. Moreover, other specific protein sequences were detected for different groups of *Burkholderia* and used as molecular markers for improving the diagnostic assay, mostly for the clinical species [94].

Rhizobia have been amongst the better-studied PGPR, due to their ability to fix nitrogen in symbiosis with legume plants, a process that researchers have been trying to understand for more than a century [95]. A taxonomic revision of the family *Rhizobiaceae*, of the phylum proteobacteria focusing on the genera *Agrobacterium, Rhizobium, Shinella* and *Ensifer*, was recently carried out based on the phylogenomic approach [96]. It has been found that the type strain of *Rhizobium giardinii* formed a distinct clade within the members of the superclade of *Sinella* and *Ensifer*. In parallel, a study using MLSA has also proposed the clarification of the taxonomic status of *Rhizobium giardinii* by transferring this taxon to a new genus [97]. Another study including 163 genomes from *Rhizobium*, *Ensife*r, *Bradyrhizobium*, *Mesorhisobium*, *Burkholderia*, *Cupriavidus, Methylobacterium*, *Azorhizobium* and *Microvirga* has analyzed their phylogenetic diversity according to their geographic localization [56]. Plant-related genes and their distribution through these genera were analyzed, and it was found that, for example, a berberine-like domain (related with pathogen defense response) is phylogenetically restricted to some groups [56].

### 6.3. Analyses in Actinobacteria

The genomes of other phyla have also been analyzed in order to locate these lifestyle-taxonomic links. Among several genus of the phylum *Actinobacteria* identified as PGPR (Table S1), the genus *Frankia* encompasses nitrogen-fixing bacteria, well known by their symbiotic association in the root nodule of diverse taxonomic actinorhizal plants [98], their saprophytic, facultative and obligate symbiont lifestyle and their PGPR effect [99],[100],[101]. The genus was structured in four groups or clades according to the host plant specificity [102]–[108]. Since the availability of whole genome sequences of *Frankia* strains cover the genetic diversity of each lineage of the genus, the lifestyle of this actinobacterium was deciphered by highlighting the clear correlation between the genome size, host-plant range and *Frankia* lifestyle [101],[109]. In fact, the genome size is highly variable between groups: (i) clade 1 varied from 7.5 Mb for strains recognized by its intermediaries” host-range to 5.4 Mb for subclade 1 of *Frankia*-*Casuarina* know by their facultative symbiont lifestyle and limited host range; (ii) clade 2 varied from 5.3 to 5.8 Mb for *Frankia* known by its low genetic variability and its facultative and non-cultivable symbiont lifestyle; (iii) clade 3 members possess 8.9 Mb, being strains associated with a broad range of host-plant and known by their saprophytic lifestyle; and (iv) clade 4 ranged from 6.7 Mb to 9.9 Mb corresponding to atypic *Frankia*, non-infective and/or non-effective, isolated from different host-plant [101].

In addition, the availability of the genome sequences of *Frankia* strains provides a significant progress at a taxonomic level and in the physiologic potential of *Frankia* in terms of natural product biosynthesis [100]. Using the genome sequence for calculating the digital DDH value between *Frankia* strains helped to complete the taxonomy of this taxon by describing a new species inside each clade [110]–[113], after more than one decade from the first description of *Frankia alni* [114],[115]. Comparative genomics of 25 *Frankia* strains revealed the presence of an unexpected number of gene bioclusters encoding for siderophore, signaling molecules [100], nitrogenase, uptake hydrogenase, hopanoid, truncated hemoglobin and stress tolerance [116]. These are later involved in symbiosis with the plants and it seems different from what has been described before, knowing that some of the genes were scattered in the genome [100],[109],[116] and not clustered. This genomic insight highlights the significant importance of this nitrogen-fixing symbiotic actinobacterium, which can be an excellent candidate for biotechnology engineering development applied in agriculture or in phytoremediation fields.

Another advantage for PGPB is the ability to cope with cold environments by the production of xeroprotectants [117],[118], as is the case in some species of *Arthrobacter* [118],[119]. Whole genome sequences have been used to confirm this feature and to provide more genetic information about the evolutionary relatedness of cold-shock protein with other proteins, such as chaperone HSP31, glyoxalase 3, S1 RNA binding protein or rhodanase, which are helpful to decipherate the mechanism of tolerance of bacteria to the temperature stress [120]. Other species of *Arthrobacter* were identified as desiccation-tolerant bacteria, such as *Arthrobacter siccitolerans* [118] and *Arthrobacter koreensis* [121]. This later feature was also found in *Rhodococcus sp* and *Leucobacter* genera [122],[123]. In this regard, whole genome sequences provide more insights about the PGP effects of the *Arthrobacter* sp., as described in Manzanera et al. [124] and Singh et al. [120].

### 6.4. Analyses in Firmicutes

Genome sequence analysis of *Bacillus* strains has also been used for understanding the molecular mechanisms of PGP at different levels; for example, deciphering genes associated with plant disease in order to have a better application of biocontrol, which consequently may have positive economic impact in the society [125]. The genus *Bacillus* is considered as one of the predominant taxons with a PGPR effect in the phylum *Firmicutes* [125]. The genus encompasses 355 species with validly published names, according to LPSN classification [91] in which the group of *Bacillus subtilis* was characterized by their stimulation of plant growth and plant anti-pathogenic effect [126],[127],[128]. Phylogenomic and phylogenetic analysis based on the average nucleotide identity (ANI) approach (calculated from pair-wise comparisons of all sequences shared between any two strains [27]), core genome, and *gyr*B gene were performed to study the inter-phylogenetic relationship of *Bacillus* species and led to clarifying the affiliation of several strains [125]. One of these strains originally classified as a member of the species *Bacillus amyloliquefaciens*, was transferred to *B. subtilis* and the type strain of the species *Bacillus siamensis* [129] was transferred to *B. amyloliquefaciens* subsp. *plantarum* [130]. This taxonomic information has a crucial importance in understanding the distribution of different subsystem categories of the genus *Bacillus*, as described in Hossain et al. [125]. Comparative genomics of members of *B. amyloliquefaciens* subsp. *amyloliquefaciens* and *B. amyloliquefaciens* subsp. *plantarum* showed 73 genes present in almost all the strains of *B. amyloliquefaciens subsp plantarum* and which are likely to also be involved in carbon degradation and signaling between others [125]. These genes could be responsible for the interaction of members of *B. amyloliquefaciens* subsp. *plantarum* with plant and rhizosphere [125]. The same authors have confirmed the high potential biocontrol activities of *B. amyloliquefaciens* subsp. *plantarum* strains after detection of genes encoding for difficin (*dfn*D) and macrolactin.

### 6.5. Analyses in Bacteroidetes

Members of the genus *Flavobacter* (phylum *Bacteroidetes*) have commanded the attention of the scientific community in different fields due to their ecological heterogeneity (aquatic and terrestrial habitats). Moreover, they belong to completely distinct categories, as some species are pathogenic to fish [131],[132] while others have been identified as PGP bacteria [133],[134], with high potential to be applied in bioremediation of terrestrial and marine soils [135],[136]. It has been shown that *Flavobacter* with terrestrial origin are ubiquitous in the rhizosphere and phyllosphere [137],[138],[139]. To understand their habitat-adaptation, mainly their abundance in the rhizosphere, a comparative genomic study of root-associated *Flavobacter* with other strains from the same genus has been carried out by Kolton et al. [42]. They found that (i) the size of the genome of *Flavobacter* varied by almost two-fold in the size between the terrestrial ones (largest genome) and the aquatic strains [42]; (ii) these two groups of *Flavobacter* (aquatic and terrestrial) formed two distinct clades based on functional similarity, and they are characterized by high number of genes implicated in carbohydrates metabolism which can be related to the adaptation of *Flavobacter* to the plant (terrestrial *Flavobacter*), and by high value of peptide and proteins (aquatic *Flavobacter*) which can led to better understanding of the lifestyle of the aquatic *Flavobacter* [42].

## 7. Future and Perspectives

Recently developed technologies in genomics and metagenomics have completely changed our vision of the microbial world. The identification of each individual sequence within a microbial community, and its classification using taxonomic tools, allows for access to basic information about their physiology, epidemiology and evolutionary history [140], obtaining indirect information about their ecological role [141]. However, how we should use this information in taxonomy is still unclear.

In microbiology, there are minimal standards for valid publication of bacterial names in microbiological journals, with criteria that prevents many of the new species descriptions be validated, resulting in literature full of names with uncertain meanings [29]. The availability of genomic information at prices even cheaper than many phenotypic tests has driven a new controversial proposal: should new classification be based on genomic information alone? Taxonomists and other researchers related to the topic have expressed opposing arguments for continually using the polyphasic taxonomy. Those against deem that retaining this methodology is an attempt to keep species descriptions as the privilege of only a small group of laboratories which are able to carry on the phenotypic, genotypic and chemotaxonomic analyses necessary [45],[142]. On the other hand, pro-polyphasic taxonomy groups remember the importance of this phenotypic characterization not only in taxonomy but also for other applications to the broader community [3],[143]. At this moment what is certain is that the scientific community is asking for changes. We should revise the utility of some of the classical techniques in these times and be permitted to incorporate all the genomic information available to generate new “minimal standards” in taxa description.

Within plant probiotics, next-generation sequencing analyses has allowed for a huge increase in our general knowledge and understanding of bacterial compositions and their abilities relative to the plants. Different methods have been proposed in phylogenomic analysis, showing in most cases clear improvement regarding classical gene phylogenies, but a deep comparison between them is necessary to define which is the best of them and should be used systematically in taxonomy. General improvements in genome accuracy are also expected for next years, as well as the development of the so-called “third generation sequencing methods” and their advantages [143],[144].

Metagenomic analyses have exposed the high diversity that inhabits plant tissues and their surroundings, including many bacteria that were not previously described as plant probiotics or else belong to new genus previously unknown. All of these have the potential to be objects of interest in the future, particularly in trying to establish their functions and their relationships with the plants. Determining the healthiest microbial composition for each crop will help to improve the agricultural system with limited use of unfriendly-environmental chemicals.

The analysis of whole genome sequences has been shown to be of great value in the redefinition of phylogenetic position and the taxonomic classification of previously unclassified or misclassified taxa [140],[145]. Moreover, important changes can be expected within this area of study in the next few years, as soon as new genomic information becomes available, and plant probiotic bacteria taxonomy will not be an exception. Indeed, taxonomic analysis and the correct determination of the bacteria that are planned to be used as probiotics in plants should be accurately carried out. If we only take into account the promotion capacity or other characteristics after isolation, without paying attention to their correct identification, we are risking including certain opportunistic pathogens into the food chain. Several bacteria belonging to *Achromobacter*, *Enterobacter*, *Erwinia*, *Ochrobactrum*, *Paenibacillus, Pseudomonas, Serratia* or *Stenotrophomonas* genera have been previously described as probiotics of plants. However, as many species of these genera are considered as human potential pathogens, special attention should be paid in their identification. New-omics technologies have increased our capacity of identification and analysis of plant probiotic microorganisms in an unprecedented manner; however, more genomic databases are needed, and the capacity of analysis of these data still has ample scope for improvement.
