# Supplementary material for: Taxonomy and systematics of plant probiotic bacteria in the genomic era
Source: AIMS Microbiol. 2017 May 31;3(3):383–412. doi: 10.3934/microbiol.2017.3.383 (PMC6604993; doi:10.3934/microbiol.2017.3.383)
Supplement: Supplementary file 1 [file microbiol-03-03-383-s001.pdf]

AIMS Microbiology

AIMS Microbiology, 3(3): 383-412.
DOI: 10.3934/microbiol.2017.3.383
Received: 03 March 2017
Accepted: 22 May 2017
Published: 31 May 2017

http://www.aimspress.com/journal/microbiology

Review

Taxonomy and systematics of plant probiotic bacteria in the genomic era

Lorena Carro * and Imen Nouioui

School of Biology, Newcastle University, Newcastle upon Tyne, UK

* **Correspondence:** Email: lcg@usal.es.

Table S1. Some taxonomic analysis of probiotic plant genera studies.

Genera	Analysis	Plant	References
Phylum Actinobacteria			
Agromyces	ARDRA	*Bolboschoenus maritimus, Puccinella limosa, Aster tripolium*	Borsodi et al., 2015 [180]
Arthrobacter	16S rRNA and recA genes, chemotaxonomy	-	Busse, 2016 [181]
Curtobacterium	RFLP	grass	Behrendt et al., 2002 [182]
	ARDRA, rep-PCR	-	Agarkova et al., 2012 [183]
	BOX-PCR	*Tylosema esculentum*	Chimwamurombe et al., 2016 [184]
Frankia	MLSA	*Allocasuarina verticillara, Alnus crispa, Alnus rubra, Alnus viridis, Casuarina cunninghamiana, Casuarina equisetifolia, Casuarina glauca, Ceanothus americanus, Colletia hystrix, Comptonia peregrina, Coriaria arbórea, Coriaria japónica, Coriaria myrtifolia, Coriaria nepalensis, Datisca cannabina, Datisca glomerata, Discaria chacaye, Discaria trinervis, Elaeagnus angustifolia, Elaeagnus umbelata, Purshia tridentata, Morella califórnica, Morella pennsylvanica*	Nouioui et al., 2011 [107]
	MLSA, Phylogenomics	-	Sen et al., 2014 [24]

	Phylogenomics	-	Tisa et al., 2016 [185]
Microbacterium	ERIC-PCR, MLSA	*Halimione portulacoides*	Fidalgo et al., 2016 [186]
	ARDRA	*Triticum aestivum*	Landa et al., 2003 [187]
	BOX-PCR	*Tylosema esculentum*	Chimwamurombe et al., 2016 [184]
Micromonospora	MLSA, ARDRA	*Pisum sativum*	Carro et al. 2012 [12]
	BOX-PCR	*Lupinus angustifolia*	Trujillo et al., 2010 [13]
Streptomyces	MLSA	-	Rong & Huang, 2012 [188]
	RFLP	*Triticum aestivum*	Conn & Franco, 2004 [189]
Rhodococcus	ARDRA	*Hordeum vulgare,* weed	Kim et al., 2011 [148]
Phylum Bacteroidetes			
Flavobacterim	Phylogenomics	-	Zeng et al., 2015 [190]
	MLSA	-	Ashrafi et al., 2015 [191]
	ARDRA	*Triticum aestivum*	Landa et al., 2003 [187]
Chryseobacterium	ARDRA	*Triticum aestivum*	Landa et al., 2003 [187]
	RFLP	*Zingiber officinale*	Chen et al., 2014 [192]
Pedobacter	Phylogenomics	-	Hahnke et al., 2016 [145]
Sphingobacterium	ARDRA	*Nicotiana tabacum*	Tian et al., 2009 [193]
Phylum Firmicutes			
Bacillus	ARDRA	*Allium cepa, Allium fistulosum, Allium sativum, Brassica, Hordeum vulgare,* weed	Kim et al., 2011 [148]
	RFLP	*Zingiber officinale*	Chen et al., 2014 [192]
	RAPD, MLSA	*Triticum aestivum, Oryza sativa, Medicago sativa*	Mohkam et al., 2016 [194]
	RFLP, MLSA	*Prosopis farcta*	Fterich et al., 2011 [195]
	BOX-PCR	*Tylosema esculentum*	Chimwamurombe et al., 2016 [184]
Brevibacillus	ARDRA	Weed	Kim et al., 2011 [148]
	BOX-PCR, ARDRA	*Oryza sativa*	Yang et al., 2008 [196]
Paenibacillus	ARDRA	*Allium fistulosum, Brassica napa, Hordeum vulgare,* weed	Kim et al., 2011 [148]
	RFLP, MLSA	*Prosopis farcta*	Fterich et al., 2011 [195]
	BOX-PCR	*Capsicum annuum*	Phi et al., 2010 [197]
Sporosarcina	ARDRA	Weed	Kim et al., 2011 [148]
Terribacillus	BOX-PCR	-	Ettoumi et al., 2013 [198]
Viridibacillus	ARDRA	Weed	Kim et al., 2011 [148]
	BOX-PCR, Chemotaxonomy	-	Albert et al., 2007 [199]

Phylum Proteobacteria			
Acetobacter	ARDRA	Weed	Kim et al., 2011 [148]
	RAPD, ARDRA, MLSA	-	Li et al., 2014 [200]
Achromobacter	MLSA	-	Gomila et al., 2014 [201]
Acinetobacter	ARDRA	*Allium cepa*	Kim et al., 2011 [148]
	RFLP, MLSA	*Prosopis farcta*	Fterich et al., 2011 [195]
	RAPD	*Saccharum officinarum*	Velázquez et al., 2008 [202]
Aeromonas	MLSA	-	Roger et al., 2012 [203]
Agrobacterium	MLSA	-	Mousavi et al., 2015 [97]
	ARDRA	*Eucalyptus sp.*	Procópio et al., 2009 [204]
Azospirillum	RAPD, ARDRA	*Saccharum officinarum, Zea mays, Manihot esculenta*	Reinhardt et al., 2008 [205]
Azorhizobium	RFLP	Sesbania rostrata	Capoen et al., 2007 [206]
Azotobacter	RAPD, BOX-PCR	-	Lenart-Boron et al., 2014 [207]
Bradyrhizobium	MLSA	*Ornithopus compressus*	Rivas et al., 2009 [208]
	RAPD, MLSA	*Cytisus villosus*	Ahnia et al., 2014 [209]
Burkholderia	ARDRA	Weed	Kim et al., 2011 [148]
	BOX-PCR	*Tylosema esculentum*	Chimwamurombe et al., 2016 [184]
	MLSA	-	Ong et al., 2016 [210]
	Phylogenomics	-	Peeters et al., 2016 [211]
Devosia	RAPD, RFLP	*Neptunia natans*	Rivas et al., 2003 [212]
Ensifer	ARDRA	Weed	Kim et al., 2011 [148]
	MLSA	-	Martens et al., 2008 [22]
	RFLP, MLSA	*Prosopis farcta*	Fterich et al., 2011 [195]
Enterobacter	MLSA	*Nicotiana tabacum*	Duan et al., 2015 [213]
Erwinia	MLSA, Phylogenomics	-	Facey et al., 2015 [214]
Herbaspirillum	RAPD, ARDRA	*Saccharum officinarum, Zea mays, Manihot esculenta*	Reinhardt et al., 2008 [205]
Janthinobacterium	RFLP	*"Pinus silvestris"*	Izumi et al., 2006 [215]
Kosakonia	BOX-PCR	*Tylosema esculentum*	Chimwamurombe et al., 2016 [184]
	MLSA	*Zea mays*	Kämpfer et al., 2016[216]
Mesorhizobium	BOX-PCR	*Caragana arborescens*	Lu et al., 2009 [217]
	RFLP, MLSA	*Prosopis farcta*	Fterich et al., 2011 [195]
	MLSA	*Cicer canariense*	Pérez-Yépez et al., 2014 [16]
Methylobacterium	BOX-PCR	*Tylosema esculentum*	Chimwamurombe et al., 2016 [184]
	ARDRA	*Crotalaria sp.*	Sy et al., 2001 [218]
Methylotropic	ARDRA	Weed	Kim et al., 2011 [148]
Novosphingobium	RFLP	-	Tiirola et al., 2002 [219]

	phylogenomics	-	Aylward et al., 2013 [220]
Pantoea	ARDRA	*Allium fistulosum*	Kim et al., 2011 [148]
	BOX-PCR	*Tylosema esculentum*	Chimwamurombe et al., 2016 [184]
	MLSA	*Eucalyptus sp.*	Brady et al., 2012 [221]
	Phylogenomics	*Frankliniella occidentalis*	Facey et al., 2015 [214]
Phyllobacterium	ARDRA, RFLP, BOX-PCR	*Vicia sp.*	Lei et al., 2008 [222]
Pseudomonas	ARDRA	*Triticum aestivum*	Landa et al., 2003 [187]
	ARDRA	*Allium cepa, Allium fistulosum, Brassica napa, Hordeum vulgare,* weed	Kim et al., 2011 [148]
	RAPD, RFLP	*Cicer arietinum*	Singh, 2015 [14]
	MLSA	-	Mulet et al., 2010 [223]
	ARDRA, BOX-PCR, ERIC-PCR	*Mentha piperita*	Santoro et al., 2016 [175]
	Phylogenomics		
Ochrobactrum	RAPD, ARDRA	*Oryza sativa*	Tripathi et al., 2002 [224]
Ralstonia	ARDRA	*Triticum aestivum*	Landa et al., 2003 [187]
	Phylogenomics	-	Zhang & Qiu, 2016 [225]
Ranhella	ARDRA	Weed	Kim et al., 2011 [148]
	MLSA	*Quercus sp.*	Brady et al., 2014 [226]
Rhizobium	MLSA	-	Mousavi et al., 2015 [97]
	BOX-PCR	*Tylosema esculentum*	Chimwamurombe et al., 2016 [184]
	Phylogenomics	-	Ormeño-Orillo et al., 2015 [96]
Serratia	ARDRA	*Allium fistulosum*	Kim et al., 2011 [148]
Stenotrophomonas	BOX, ERIC and REP-PCR	-	
	ARDRA	*Hordeum vulgare*	Kim et al., 2011 [148]
	MLSA	-	Ramos et al., 2011 [227]
Staphylococcus	MLSA	-	Margos et al., 2012 [228]
Variovorax	ARDRA	Weed	Kim et al., 2011 [148]

© 2017 Lorena Carro, et al., licensee AIMS Press. This is an open access article distributed under the terms of the Creative Commons Attribution License (http://creativecommons.org/licenses/by/4.0)
